# Supplementary material for: Morphological variation suggests that chitinozoans may be fossils of individual microorganisms rather than metazoan eggs
Source: Proc Biol Sci. 2019 Jul 31;286(1908):20191270. doi: 10.1098/rspb.2019.1270 (PMC6710598; doi:10.1098/rspb.2019.1270)
Supplement: Measurements on Hercochitina violana sp. nov. and randomly selected chitinozoans, the egg size variation in extant invertebrates and its selecting criteria. [file rspb20191270supp1.pdf]

Article title: Morphological variation suggests that chitinozoans may be fossils of individual microorganisms rather than metazoan eggs

Authors: Liang, Yan; Bernardo, Joseph; Goldman, Daniel; Nõlvak, Jaak; Tang, Peng ; Wang, Wenhui; Hints, Olle

Journal name: *Proceedings of the Royal Society B: Biological Sciences*

Article DOI: 10.1098/rspb.

Electronic supplementary material §1. Measurements on *Hercochitina violana* sp. nov.

Measurement on 331 specimens from two samples. Data are presented in ascending order of the vesicle length.

Specimen	L	D_p	D_{cons}	L/D_p	D_{cons}/D_p
37.8 1	93.01	/	/	/	/
43-1-3	94.05	71.35	51.35	1.32	0.72
43-1-2	98.92	88.65	74.59	1.12	0.84
43-1-1	102.16	78.92	62.16	1.29	0.79
sem- 2	106.27	92.62	67.53	1.15	0.73
43-1-4	107.03	88.11	60.54	1.21	0.69
37.8 2	108.06	54.84	40.32	1.97	0.74
43-1-12	110.81	66.49	53.51	1.67	0.80
43-1-11	112.43	64.32	47.57	1.75	0.74
sem- 1	114.02	100.37	85.24	1.14	0.85
37.8 3	116.13	74.73	59.14	1.55	0.79
43-1-5	117.30	89.19	60.00	1.32	0.67
43-1-9	120.00	104.86	83.24	1.14	0.79
43-1-8	121.08	103.24	78.38	1.17	0.76
43-1-13	122.70	68.11	51.35	1.80	0.75
sem- 3	123.99	84.50	70.85	1.47	0.84
sem- 4	124.72	78.60	63.10	1.59	0.80
37.8 5	124.73	75.81	58.60	1.65	0.77

37.8 4	126.34	66.13	56.45	1.91	0.85
43-1-17	127.03	65.95	48.65	1.93	0.74
37.8 6	127.42	70.97	46.77	1.80	0.66
43-1-6	128.11	75.14	61.62	1.71	0.82
43-1-14	131.35	61.08	44.86	2.15	0.73
43-1-18	131.89	65.41	51.89	2.02	0.79
43-1-7	132.43	89.73	67.03	1.48	0.75
sem- 6	135.42	78.23	67.53	1.73	0.86
43-1-16	135.68	70.81	55.14	1.92	0.78
43-1-15	135.68	66.49	54.59	2.04	0.82
37.8 15	136.02	79.57	61.83	1.71	0.78
43-1-22	136.22	60.54	48.65	2.25	0.80
37.8 10	137.10	80.11	64.52	1.71	0.81
37.8 7	137.10	63.98	52.15	2.14	0.82
37.8 9	137.10	/	52.15	/	/
43-1-19	137.30	71.89	52.43	1.91	0.73
43-1-10	138.92	97.84	70.81	1.42	0.72
sem- 5	139.11	72.69	55.72	1.91	0.77
43-1-20	139.46	89.73	70.27	1.55	0.78
37.8 8	141.94	74.73	60.75	1.90	0.81
43-1-66	142.16	77.30	60.54	1.84	0.78
37.8 20	142.47	75.81	57.53	1.88	0.76
43-1-60	142.70	84.32	61.62	1.69	0.73
43-1-25	142.70	74.05	59.46	1.93	0.80
43-1-68	144.32	79.46	60.54	1.82	0.76
43-3-1	144.86	77.30	61.08	1.87	0.79
43-1-21	144.86	76.76	57.84	1.89	0.75
37.8 13	145.70	80.11	61.29	1.82	0.77
43-1-23	145.95	83.78	62.70	1.74	0.75
sem- 7	146.13	92.99	74.54	1.57	0.80
37.8 23	146.24	83.33	63.44	1.75	0.76
43-1-31	146.49	74.05	51.35	1.98	0.69
37.8 11	147.31	88.71	65.59	1.75	0.81
37.8 19	147.85	72.04	51.08	2.05	0.71
37.8 24	147.85	83.87	64.52	1.76	0.77
43-1-64	148.11	70.81	52.43	2.09	0.74
43-1-59	148.11	75.14	62.16	1.97	0.83
37.8 21	148.39	71.51	59.14	2.08	0.83
43-1-63	149.73	85.41	74.05	1.75	0.87
43-3-54	151.35	83.78	56.22	1.81	0.67
43-2-23	152.43	84.86	64.86	1.80	0.76
43-1-24	153.51	74.59	/	2.06	/

43-1-38	154.05	78.38	54.05	1.97	0.69
43-1-33	154.05	78.92	65.95	1.95	0.84
43-1-42	154.05	78.38	61.08	1.97	0.78
37.8 12	154.30	85.48	64.52	1.81	0.75
43-1-27	154.59	80.00	61.08	1.93	0.76
43-1-35	154.59	92.97	72.97	1.66	0.78
43-4-1	155.14	88.11	65.41	1.76	0.74
43-1-70	155.14	83.78	62.70	1.85	0.75
sem- 8	156.46	73.06	56.83	2.14	0.78
43-3-2	156.76	79.46	56.76	1.97	0.71
43-2-24	156.76	90.81	60.54	1.73	0.67
43-1-71	157.30	83.78	64.86	1.88	0.77
43-1-62	157.30	89.19	77.84	1.76	0.87
37.8 18	157.53	69.89	60.75	2.25	0.87
43-3-4	157.84	75.14	50.81	2.10	0.68
43-1-26	157.84	75.14	61.62	2.10	0.82
43-1-49	157.84	89.19	65.95	1.77	0.74
43-1-65	157.84	84.86	68.65	1.86	0.81
37.8 25	158.06	80.11	66.13	1.97	0.83
43-1-28	158.38	87.03	64.86	1.82	0.75
37.8 28	158.60	83.87	60.22	1.89	0.72
43-1-67	158.92	81.08	62.16	1.96	0.77
43-1-30	158.92	80.54	58.92	1.97	0.73
43-1-32	158.92	81.08	65.41	1.96	0.81
43-1-61	158.92	97.84	73.51	1.62	0.75
37.8 14	159.14	70.43	58.06	2.26	0.82
43-1-34	159.46	78.92	64.32	2.02	0.82
37.8 22	159.68	72.58	59.68	2.20	0.82
37.8 16	159.68	72.58	61.83	2.20	0.85
43-2-25	160.54	85.95	64.86	1.87	0.75
37.8 17	160.75	73.66	59.14	2.18	0.80
43-3-3	161.08	85.41	56.76	1.89	0.66
43-3-10	161.08	78.38	60.00	2.06	0.77
43-3-12	162.16	79.46	51.89	2.04	0.65
43-1-40	162.16	83.24	63.24	1.95	0.76
43-1-39	162.16	87.03	67.03	1.86	0.77
43-2-26	162.70	87.57	66.49	1.86	0.76
43-2-27	163.24	82.16	62.16	1.99	0.76
sem- 9	163.84	78.97	53.87	2.07	0.68
43-3-9	164.32	72.97	58.38	2.25	0.80
43-1-43	164.32	92.97	64.86	1.77	0.70
43-1-51	164.32	78.92	61.08	2.08	0.77

43-1-29	164.86	83.78	72.97	1.97	0.87
43-1-48	164.86	84.32	63.24	1.96	0.75
43-1-72	165.41	80.00	49.73	2.07	0.62
43-1-46	165.95	71.89	61.08	2.31	0.85
43-3-8	165.95	86.49	63.24	1.92	0.73
43-3-5	165.95	81.08	60.54	2.05	0.75
43-1-69	165.95	82.70	64.32	2.01	0.78
43-1-37	165.95	81.08	67.57	2.05	0.83
43-4-3	166.49	78.38	61.62	2.12	0.79
43-1-44	166.49	91.35	72.43	1.82	0.79
43-1-57	167.03	78.38	62.16	2.13	0.79
43-3-7	167.57	89.73	63.24	1.87	0.70
37.8 35	167.74	96.24	73.66	1.74	0.77
43-3-11	168.11	75.14	60.54	2.24	0.81
43-1-45	168.65	77.30	54.59	2.18	0.71
43-1-36	169.19	87.03	56.76	1.94	0.65
43-1-58	170.27	80.54	63.24	2.11	0.79
37.8 29	170.43	76.34	55.91	2.23	0.73
43-3-26	170.81	81.08	63.78	2.11	0.79
43-1-52	171.35	76.22	61.08	2.25	0.80
37.8 36	171.51	79.57	53.23	2.16	0.67
43-1-47	171.89	79.46	55.68	2.16	0.70
43-1-41	171.89	82.16	64.32	2.09	0.78
43-3-13	171.89	82.16	71.35	2.09	0.87
43-2-4	171.89	90.27	67.57	1.90	0.75
43-2-3	171.89	87.57	67.03	1.96	0.77
37.8 30	172.04	82.80	65.59	2.08	0.79
43-3-24	172.43	91.89	80.00	1.88	0.87
43-3-6	172.43	89.19	65.41	1.93	0.73
43-1-73	172.97	88.11	70.27	1.96	0.80
43-2-30	174.05	82.16	64.32	2.12	0.78
43-1-54	174.05	85.41	62.70	2.04	0.73
43-2-11	174.05	82.16	62.70	2.12	0.76
43-3-27	174.05	82.16	63.24	2.12	0.77
43-2-28	174.59	79.46	52.43	2.20	0.66
43-3-30	174.59	71.89	60.00	2.43	0.83
37.8 26	174.73	82.80	60.75	2.11	0.73
43-1-75	175.14	85.95	69.73	2.04	0.81
37.8 34	175.27	87.63	71.51	2.00	0.82
43-2-29	176.22	90.81	64.86	1.94	0.71
sem- 11	176.75	85.98	60.89	2.06	0.71
43-4-4	177.30	75.68	56.76	2.34	0.75

43-3-23	177.30	80.00	59.46	2.22	0.74
43-1-50	177.30	83.24	62.70	2.13	0.75
37.8 27	177.42	91.94	69.89	1.93	0.76
43-3-29	177.84	116.22	82.70	1.53	0.71
43-2-14	177.84	89.19	70.81	1.99	0.79
43-2-5	177.84	90.81	72.97	1.96	0.80
43-1-53	177.84	82.70	69.73	2.15	0.84
37.8 33	177.96	87.63	63.44	2.03	0.72
37.8 40	177.96	99.46	/	1.79	/
43-3-34	178.38	85.41	69.19	2.09	0.81
37.8 32	178.49	72.58	55.91	2.46	0.77
37.8 31	178.49	92.47	70.97	1.93	0.77
43-2-2	178.92	90.27	69.19	1.98	0.77
43-3-25	178.92	84.32	64.86	2.12	0.77
43-1-92	178.92	71.89	56.76	2.49	0.79
43-3-28	179.46	89.19	64.32	2.01	0.72
43-3-31	180.54	89.19	74.59	2.02	0.84
43-4-5	180.54	77.84	60.00	2.32	0.77
43-1-83	181.08	84.86	/	2.13	/
43-4-2	181.62	82.70	56.22	2.20	0.68
43-1-93	182.16	85.41	60.54	2.13	0.71
43-1-77	182.70	92.97	73.51	1.97	0.79
43-1-74	182.70	91.35	72.97	2.00	0.80
43-1-94	182.70	80.54	66.49	2.27	0.83
43-4-7	182.70	88.65	61.62	2.06	0.70
43-2-16	182.70	83.78	67.57	2.18	0.81
43-2-6	183.24	89.19	62.16	2.05	0.70
43-4-17	183.24	81.08	59.46	2.26	0.73
sem- 10	183.39	87.45	71.22	2.10	0.81
43-1-76	183.78	74.59	58.38	2.46	0.78
43-4-16	184.32	90.27	60.54	2.04	0.67
43-3-32	184.32	82.70	70.81	2.23	0.86
43-3-22	184.32	76.22	62.16	2.42	0.82
43-3-21	184.32	97.84	71.89	1.88	0.73
37.8 39	184.41	78.49	59.68	2.35	0.76
43-3-15	184.86	82.16	57.84	2.25	0.70
43-1-56	184.86	87.57	65.41	2.11	0.75
43-4-15	185.41	87.03	56.22	2.13	0.65
43-1-80	185.41	87.57	61.62	2.12	0.70
43-2-33	185.95	87.57	64.32	2.12	0.73
43-2-32	185.95	77.84	57.30	2.39	0.74
37.8 38	186.02	90.32	68.28	2.06	0.76

43-1-91	186.49	75.68	58.38	2.46	0.77
43-3-33	186.49	78.38	62.70	2.38	0.80
43-3-35	186.49	101.08	75.14	1.84	0.74
43-2-7	187.03	89.73	63.24	2.08	0.70
43-3-49	187.57	91.35	63.24	2.05	0.69
43-1-89	187.57	101.62	75.68	1.85	0.74
43-3-20	187.57	75.68	56.22	2.48	0.74
43-4-19	187.57	82.16	63.24	2.28	0.77
43-4-6	188.11	85.41	59.46	2.20	0.70
43-2-31	188.65	88.65	69.19	2.13	0.78
43-3-42	188.65	85.41	67.03	2.21	0.78
43-3-16	188.65	83.78	68.65	2.25	0.82
43-1-90	189.19	85.95	63.78	2.20	0.74
43-2-10	189.19	95.68	71.89	1.98	0.75
43-4-8	189.19	94.59	75.68	2.00	0.80
37.8 37	189.25	93.55	72.04	2.02	0.77
43-2-15	189.73	84.86	54.05	2.24	0.64
43-1-85	189.73	95.68	71.35	1.98	0.75
43-4-13	190.27	87.03	67.03	2.19	0.77
43-3-48	190.27	89.73	69.19	2.12	0.77
43-2-20	190.81	89.73	68.11	2.13	0.76
43-2-8	190.81	75.68	57.84	2.52	0.76
37.8 46	190.86	91.94	78.49	2.08	0.85
sem- 12	191.51	88.93	68.63	2.15	0.77
43-3-37	191.89	78.38	58.38	2.45	0.74
43-4-10	191.89	78.92	64.86	2.43	0.82
43-1-55	191.89	90.27	64.86	2.13	0.72
43-4-18	192.43	84.86	67.57	2.27	0.80
43-4-9	192.43	98.38	72.43	1.96	0.74
37.8 41	192.47	83.33	67.20	2.31	0.81
43-1-86	192.97	88.11	69.19	2.19	0.79
43-4-11	193.51	/	/	/	/
43-2-43	194.05	80.00	57.30	2.43	0.72
43-4-12	194.05	76.76	56.22	2.53	0.73
43-2-44	194.59	92.43	63.78	2.11	0.69
37.8 43	194.62	99.46	67.20	1.96	0.68
43-1-84	195.14	87.03	64.32	2.24	0.74
43-2-1	195.68	80.00	/	2.45	/
43-3-53	195.68	92.43	69.19	2.12	0.75
37.8 42	195.70	83.33	63.44	2.35	0.76
43-2-18	196.22	87.57	60.54	2.24	0.69
43-3-39	196.76	101.62	74.59	1.94	0.73

43-3-36	196.76	83.78	63.24	2.35	0.75
43-2-21	196.76	82.70	64.86	2.38	0.78
43-2-19	196.76	91.89	67.57	2.14	0.74
43-2-9	196.76	84.32	60.00	2.33	0.71
43-3-17	196.76	101.08	78.38	1.95	0.78
43-3-38	197.30	87.57	61.62	2.25	0.70
43-3-40	197.30	84.86	63.24	2.32	0.75
43-2-36	197.30	85.41	63.78	2.31	0.75
43-3-43	197.84	92.97	69.19	2.13	0.74
43-2-13	197.84	81.62	62.70	2.42	0.77
43-4-14	197.84	80.54	62.16	2.46	0.77
43-2-41	197.84	82.16	65.95	2.41	0.80
43-3-14	198.92	83.78	59.46	2.37	0.71
43-3-18	198.92	84.32	62.70	2.36	0.74
43-4-22	198.92	89.73	70.81	2.22	0.79
43-2-35	198.92	85.41	68.65	2.33	0.80
43-1-87	199.46	88.65	64.86	2.25	0.73
43-2-34	199.46	90.81	69.73	2.20	0.77
sem- 13	199.63	85.61	70.48	2.33	0.82
43-2-12	200.00	83.24	61.62	2.40	0.74
43-3-19	200.54	93.51	66.49	2.14	0.71
43-3-47	200.54	85.41	61.08	2.35	0.72
43-3-50	201.62	86.49	69.73	2.33	0.81
43-1-81	202.16	96.22	75.14	2.10	0.78
43-2-38	202.70	92.97	69.73	2.18	0.75
43-3-51	202.70	89.19	69.19	2.27	0.78
43-2-17	202.70	87.57	68.65	2.31	0.78
sem- 15	202.95	91.14	77.12	2.23	0.85
43-2-40	203.24	89.73	55.68	2.27	0.62
43-3-41	203.78	97.30	67.57	2.09	0.69
43-2-22	203.78	88.11	70.81	2.31	0.80
37.8 44	204.30	80.11	59.14	2.55	0.74
43-1-88	204.32	97.84	70.27	2.09	0.72
43-2-39	204.86	102.70	69.73	1.99	0.68
43-3-52	205.41	92.43	66.49	2.22	0.72
43-3-45	205.95	94.59	74.05	2.18	0.78
43-2-42	205.95	93.51	71.89	2.20	0.77
43-2-37	206.49	87.57	/	2.36	/
37.8 45	206.99	90.86	75.81	2.28	0.83
43-4-28	209.19	90.81	70.27	2.30	0.77
43-4-23	209.19	91.35	65.41	2.29	0.72
43-3-44	209.73	90.27	66.49	2.32	0.74

43-4-26	210.27	98.38	71.89	2.14	0.73
sem- 16	211.81	88.93	70.48	2.38	0.79
sem- 17	211.81	97.42	80.07	2.17	0.82
43-4-21	211.89	97.30	71.35	2.18	0.73
43-4-25	212.97	91.35	67.57	2.33	0.74
43-4-24	213.51	91.89	70.81	2.32	0.77
43-1-82	214.05	94.59	64.86	2.26	0.69
43-4-20	216.22	90.27	70.81	2.40	0.78
43-4-29	216.22	101.08	70.27	2.14	0.70
43-3-46	216.76	90.27	68.11	2.40	0.75
43-4-31	218.92	99.46	67.57	2.20	0.68
43-3-55	219.46	90.81	76.76	2.42	0.85
sem- 19	221.40	97.42	74.17	2.27	0.76
43-4-27	221.62	87.03	65.41	2.55	0.75
sem- 21	222.14	92.62	73.43	2.40	0.79
sem- 18	222.88	97.42	73.43	2.29	0.75
sem- 20	224.35	93.73	76.38	2.39	0.81
43-3-56	227.57	90.81	71.35	2.51	0.79
43-2-48	229.73	90.27	65.41	2.54	0.72
43-2-47	230.27	98.92	72.43	2.33	0.73
43-3-59	230.27	105.41	83.24	2.18	0.79
43-4-30	230.81	101.08	75.68	2.28	0.75
43-3-57	232.43	96.22	73.51	2.42	0.76
43-3-58	234.05	104.86	74.59	2.23	0.71
43-4-33	235.68	97.30	72.97	2.42	0.75
43-2-45	235.68	99.46	78.38	2.37	0.79
37.8 47	239.78	/	75.27	/	/
sem- 22	239.85	114.02	92.99	2.10	0.82
sem- 23	240.22	102.58	78.23	2.34	0.76
sem- 24	240.59	95.94	77.12	2.51	0.80
43-4-35	241.62	95.68	64.86	2.53	0.68
43-3-61	242.16	88.65	64.32	2.73	0.73
43-2-49	242.16	109.19	81.62	2.22	0.75
43-2-46	242.70	94.05	69.73	2.58	0.74
43-4-32	243.78	93.51	68.11	2.61	0.73
43-3-62	245.95	109.19	82.16	2.25	0.75
43-3-60	247.57	96.76	71.35	2.56	0.74
43-4-34	248.11	105.41	73.51	2.35	0.70
43-4-38	248.65	111.89	84.86	2.22	0.76
43-4-37	249.19	92.97	64.86	2.68	0.70
sem- 25	250.18	105.54	76.01	2.37	0.72
43-4-36	250.81	101.08	78.92	2.48	0.78

43-2-55	253.51	88.65	63.78	2.86	0.72
43-3-63	253.51	107.57	78.38	2.36	0.73
43-2-50	254.05	103.78	74.59	2.45	0.72
43-2-52	254.59	102.70	74.59	2.48	0.73
43-2-53	255.14	103.24	73.51	2.47	0.71
43-3-64	258.38	101.08	72.43	2.56	0.72
43-3-65	260.54	89.19	72.97	2.92	0.82
43-2-51	262.16	102.70	78.38	2.55	0.76
43-2-56	266.49	103.78	77.30	2.57	0.74
sem- 27	267.16	105.54	74.17	2.53	0.70
sem- 26	267.53	105.54	75.65	2.53	0.72
43-2-54	270.27	98.38	68.65	2.75	0.70
43-2-59	277.84	118.92	84.32	2.34	0.71
43-2-57	278.38	105.95	82.16	2.63	0.78
43-2-58	279.46	114.59	75.68	2.44	0.66
43-2-60	283.24	118.38	84.86	2.39	0.72
43-2-61	295.14	120.00	91.89	2.46	0.77
43-2-62	315.14	116.22	76.22	2.71	0.66
43-2-63	317.84	109.19	78.38	2.91	0.72

Electronic supplementary material §2. Measurements on randomly selected chitinozoans

A total of 593 sets of vesicle size data on 378 species, including type species of all 57 known chitinozoan genera and additional 321 species randomly selected from the literature, were compiled. Species in red colour are the type species of the corresponding genus.

Family	Genus	Species	Number of specimens measured	L_{max}/ L_{min}	D_{max}/ D_{min}	Reference
Desmochitinidae	*Calpichitina*	*scabiosa* sp. nov.	>12	1.46	1.33	(Wilson and Hedlund, 1964)
		scabiosa	25	1.50	1.58	[2]
		annulata sp. nov.	60	1.29	1.53	[3]
		bernardae sp. nov.	39	1.76	1.24	[4]
		bernardae	7	1.43	1.30	[5]
		complanata	10	/	1.31	[6]
			20	1.25	1.33	[7]
		corinnae sp. nov.	46	1.35	1.34	[8]
		gregaria sp. nov.	50	1.27	1.34	[9]
		hemsiensis sp. nov.	?	1.40	1.42	[10]
		horentis sp. nov.	15	?	1.46	[8]
		muldiensis sp. nov.	?	1.40	1.33	[10]
		opaca sp. nov.	?	1.33	1.21	
		squamosa sp. nov.	?	1.33	1.55	
	Hoegisphaera	*glabra* sp. nov.	/	/	1.18	[11]
		glabra	2	/	1.05	[12]
	Bulbochitina	*bulbosa* sp. nov.	80	2.00	2.00	[3]
		suchomastensis	5	1.22	1.38	
	Bursachitina	*bursa* sp. nov.	2	2.20	1.63	[13, 14])
		baqaensis sp. nov.	36	2.22	1.64	[15]
		basiconcava sp. nov.	17	1.69	1.56	[16]
		conica	61	2.33	2.65	[17]
		nestorae sp. nov.	20	2.06	1.82	

		umbilicata sp. nov.	66	2.38	2.40	[18]
		umbilicata	66	2.38	2.40	[19]
		krizi	25	1.50	1.24	[3]
	Desmochitina	*nodosa* sp. nov.	13	1.33	1.28	[20]
		nodosa	?	1.36	1.37	[21]
			42	1.59	1.46	[22]
		amphorea	?	1.55	1.22	[21]
		erinacea	15	1.11	1.11	
			7	1.36	1.64	[19]
			5	1.29	1.32	
			4	1.22	1.23	
			11	1.43	1.42	
			12	1.31	1.21	
		juglandiformis sp. nov.	?	1.15	1.18	[21]
		juglandiformis	10	1.40	1.32	[3]
			20	1.64	1.55	[19]
			20	1.50	1.50	
		lata	?	1.15	1.08	[21]
			50	1.51	1.49	[23]
		lecaniella	?	1.19	1.23	[21]
		minor	?	1.27	1.38	
			40	1.85	2.30	[6]
			50	1.47	1.35	[23]
			25	1.67	1.30	[2]
			10	1.44	1.52	[24]
			38	1.49	1.83	[19]
			9	1.43	1.54	
			30	1.92	1.80	
			3	1.19	1.29	
			3	1.13	1.29	
			10	1.57	1.75	
			2	1.09	2.94	
		ornensis sp. nov.	60	2.44	2.00	[3]
		ovulum	4	1.22	1.29	[19]
			12	1.33	1.53	
			12	1.17	1.08	
			22	1.35	2.00	
			14	2.00	2.50	
			5	1.92	1.62	
		piriformis sp. nov.	?	1.16	1.19	[21]
		rugosa	?	1.16	1.18	

	Ollachitina	*ingens* sp. nov.	3	1.50	1.69	[25]
	Cuticihtina	*legrandi* sp. nov.	20	1.94	1.66	[26]
		minivelata sp. nov.	53	2.17	2.15	[15]
	Pterochitina	*perivelatum* sp. nov.	/	/	/	[27]
		hymenelytrum sp. nov.	50	1.57	1.51	[23]
		hymenelytrum	30	1.18	1.25	[24]
		perivelata	40	1.76	1.37	[3]
	Armoricochitina	*ceneratiensis* sp. nov.	20	1.40	1.41	[28]
		crassicarinata sp. nov.	45	2.36	2.11	[15]
		granulifera sp. nov.	?	1.34	1.93	[29]
		gengi sp. nov.	16	2.09	1.53	[15]
		nigerica	80	2.69	1.94	[4]
		nigerica	106	2.36	1.96	[5]
		niliensis sp. nov.	8	1.12	1.14	[30]
		reticulifera	21	1.56	1.48	[19]
			38	2.06	1.91	
			9	1.56	1.42	
	Pseudoclathrochitina	*carmenchui* sp. nov.	?	1.50	/	[31]
		carmenchui	563	3.88	3.71	[32]
		sharawraensis sp. nov.	15	1.38	1.38	[33]
	Cingulochitina	*cingulata* sp. nov.	/	/	/	[27]
		convexa sp. nov.	?	1.43	1.57	[10]
		hedei sp. nov.	?	1.17	1.20	
		kolednikensis sp. nov.	50	1.60	1.40	[9]
		plusquelleci sp. nov.	40	1.60	1.47	[3]
		serrata	170	1.79	1.74	
		wronai sp. nov.	50	1.42	1.48	[9]
	Margachitina	*margaritana* sp. nov.	3	1.26	1.14	[20]
		catenaria	20	1.44	1.94	[3]
		crassipes subsp. nov.	4	1.55	1.40	
		tenuipes subsp. nov.	40	1.66	1.25	
		elegans	105	1.59	1.53	[32]
	Urnochitina	*urna* sp. nov.	/	1.17	/	[34]
	Linochitina	*erratica* sp. nov.	24?	1.27	/	[20]
		chateauneufi sp. nov.	5	1.21	1.42	[30]

		klonkensis	50	1.56	1.61	[3]
		odiosa sp. nov.	?	1.09	/	[10]
		penequadrata sp. nov.	6	1.46	1.70	[35]
		pissotensis sp. nov.	40	2.31	1.48	[3]
	Vinnalochitina	*granosa* sp. nov.	13	/	1.49	[36]
	Kalochitina	*multispinata* sp. nov.	/	1.50	/	[37]
		multispinata	10	1.33	1.25	[7]
			30	1.53	1.26	[2]
			30	1.45	1.25	[24]
	Ordochitina	*tadlaiensis sp. nov.*	40	2.10	1.64	[26]
		nevadensis sp. nov.	20	1.44	1.30	[38]
	Eisenackitina	*castor* sp. nov.	/	1.67	/	[37]
		causiata	33	2.03	1.83	[17]
		barrandei sp. nov.	70	1.83	1.44	[9]
			20	1.71	1.45	
		bohemica	60	2.52	2.49	[3]
		granosa	?	1.26	1.15	[10]
		inanulifera sp. nov.	16	1.58	1.88	[39]
		inconspicua sp. nov.	90	2.17	1.55	[40]
		inconspicua	8	1.38	1.25	[19]
		philipi sp. nov.	?	1.65	1.69	[10]
		praevininica sp. nov.	20	1.49	1.60	[38]
		rhenana	12	1.19	1.17	[3]
		ripae sp. nov.	22	2.09	1.54	[38]
		tenuis sp. nov.	21	1.26	1.26	[41]
		vininica sp. nov.	18	1.40	1.20	[38]
	Orbichitina	*vulpina* sp. nov.	33	1.67	1.64	[26]
		vulpiensis sp. nov.	33	1.67	1.64	
	Armigutta	*hillmeri* sp. nov.	1	/	/	[22]
	Salopochitina	*monterrosae* sp. nov.	60	2.00	1.60	[31]
Conochitinidae	*Euconochitina*	*conulus* sp. nov.	3	1.05	1.16	[42]
		conulus	4	1.20	2.75	[19]
		conulus with spines	more than 4	1.67	2.13	[43]
		fenxiangensis	29	1.79	1.51	[44]
		paschaensis sp. nov.	41	1.56	1.54	[45]
		paschaensis	38	1.60	1.65	[44]
		symmetrica	15	1.38	1.23	[46]

			more than 4	1.38	1.38	[47]
			45	2.03	1.52	[44]
		vulgaris sp. nov.	12	1.25	1.28	[6]
		vulgaris	42	2.36	1.77	[3]
	Conochitina	*claviformis* sp. nov.	2	1.14	1.27	[48]
		acuminata	10	1.28	1.82	[17]
		argillophila sp. nov.	?	1.21	1.33	[10]
		armifera sp. nov.	10	2.18	1.38	[49]
		baculata sp. nov.	10	1.40	1.29	
		brevis	12	1.67	1.33	[46]
		chydaea sp. nov.	45	2.63	1.68	[6]
		chydaea	23	1.90	1.82	[3]
			184	2.68	1.80	[19]
			26	2.00	1.45	
			25	1.67	1.80	
		decipiens	6	1.22	1.08	[46]
			more than 3	1.65	1.31	[47]
		dolos sp. nov.	?	1.31	1.12	[21]
		elegans	8	1.43	1.20	[6]
			82	4.43	1.55	[2]
			15	2.16	1.56	[19]
			3	1.44	1.42	
			11	2.33	1.60	
			11	2.35	2.00	
			13	2.25	1.73	
			58	1.58	1.38	[50]
		eustachensis sp. nov.	4	1.39	2.16	[51]
		flamma sp. nov.	?	1.21	1.29	[10]
		grandicula sp. nov.	more than 3	1.23	1.18	[47]
		gueddichensis sp. nov.	60	1.65	1.43	[30]
		gunriveris sp. nov.	14	1.52	1.32	[52]
		hichami sp. nov.	34	1.80	1.75	[30]
		homoclaviformis	106	2.32	2.00	[19]
			10	1.59	1.43	
		incerta	3	1.06	1.50	
			2	1.78	1.17	
			19	1.94	2.00	

			8	2.56	1.72	
		kjellstromi	15	1.57	1.38	[24]
		kryos	10	1.21	1.25	[46]
		langei	15	1.80	1.10	[53]
			10	1.59	1.26	[54]
			6	1.23	1.18	[46]
		lepida sp. nov.	3	1.18	1.10	[6]
		leptosoma sp. nov.	?	1.65	1.31	[10]
		leviscapulae sp. nov.	31	2.12	2.24	[17]
		mamilla sp. nov.	?	1.52	1.17	[10]
		mathrafalensis sp. nov.	11	1.29	1.23	[17]
		ordinaria sp. nov.	more than 6	1.69	1.25	[47]
		ordinaria	60	1.91	1.58	[55]
		parviventer sp. nov.	26	2.32	1.55	[6]
		parviventer	4	1.35	1.50	[19]
			8	1.43	1.20	
		pachycephala	20	1.22	1.29	[3]
		pervulgata	33	1.65	1.75	[55]
		plicatura sp. nov.	80	1.72	1.27	[30]
		poumoti	8	1.47	1.36	[53]
			10	1.43	1.25	[54]
			8	1.55	1.08	[46]
		primitiva	14	1.39	1.15	[19]
		proboscifera	22	3.01	1.73	[17]
		pseudocarinata	90	2.91	2.81	[3]
		raymondii sp. nov.	more than 5	1.60	1.33	[47]
		raymondii	8	1.25	1.06	[46]
		redouanei sp. nov.	60	1.89	1.36	[30]
		rotundata sp. nov.	18	2.11	2.20	[4]
		rugata sp. nov.	20	1.55	1.08	[56]
		rugata	5	1.68	1.92	[19]
		savalaensis sp. nov.	30	2.20	1.52	[57]
		scabra sp. nov.	45	1.90	1.56	[58]
		senta sp. nov.	8	1.13	1.11	[7]
		subcylindrica	5	1.14	1.36	[54]
		tigrina sp. nov.	?	1.55	1.25	[21]
		tuba	?	1.66	/	[10]
			20	1.93	1.40	[3]
		ventriosa sp. nov.	more than	1.75	1.33	[47]

			4			
		viiuae sp. nov.	15	2.23	1.97	[15]
		viruana sp. nov.	36	1.50	1.35	[57]
	Clavachitina	*claviformis* sp. nov.	5	2.28	1.36	[20, 59]
		pervulgata	more than 4	1.63	1.20	[47]
	Pistillachitina	*pistilliformis* sp. nov.	4	1.32	1.25	[60]
	Rhabdochitina	*magna* sp. nov.	/	2.00	1.25	[20]
		magna	15	1.21	1.29	[6]
			20	2.24	1.50	[61]
			20	2.00	1.50	[7]
			10	2.29	1.60	[3]
			2	1.19	1.08	[19]
			3	1.61	1.43	
		minnesotensis	30	2.08	1.44	[23]
			8	1.29	1.16	[54]
			5	1.27	1.05	[24]
			5	2.14	1.86	[19]
		curvata sp. nov.	9	1.97	2.09	[5]
		tubularis	7	1.10	1.06	[46]
		turgida sp. nov.	10	2.48	1.90	[6]
		turgida	3	1.18	/	[23]
		usitata sp. nov.	35	2.21	1.53	[6]
		usitata	7	1.61	2.19	[23]
			8	2.71	1.69	[19]
			2	1.05	1.47	
	Velatachitina	*nebulosa* sp. nov.	2	1.07	1.12	[25]
		veligera	50	2.20	1.50	[3]
	Eremochitina	*baculata* sp. nov.	3	1.18	1.19	[13, 48]
		brevis morphotype A	20	1.49	1.22	[3]
		brevis morphotype B	22	1.63	1.55	
	Siphonochitina	*formosa* sp. nov.	23	1.81	1.13	[6]
		formosa	41	2.10	1.38	[3]
			8	1.67	1.60	[19]
		clavata sp. nov.	7	1.67	1.55	[6]
		jenkinsi sp. nov.	27	1.90	1.68	[3]
		robusta sp. nov.	45	1.70	1.27	[6]
		robusta	2	1.17	1.10	[19]
			2	1.03	1.06	

		tenuicollis sp. nov.	15	1.51	1.20	[6]
	Hyalochitina	*hyalophrys* sp. nov.	/	/	/	[62]
		incomposita sp. nov.	9	1.39	1.53	[5]
	Laufeldochitina	*stentor* sp. nov.	3	1.49	1.37	[27]
		stentor	?	1.44	1.50	[21]
		agrestis sp. nov.	6	1.66	1.09	[2]
		armoricana	20	1.30	1.38	[3]
		lardeuxi sp. nov.	16	1.43	1.36	
		martinae sp. nov.	20	1.51	1.68	
		striata	10	2.14	2.05	[63]
	Baltochitina	*sagenachitina* sp.	1	/	/	[29]
		delicata sp. nov.	18	1.67	1.29	[64]
	Tanuchitina	*ontariensis* sp. nov.	?	1.40	/	[37]
		anticostiensis sp. nov.	4	2.14	1.38	[61]
		anticostiensis	10	4.00	1.31	[49]
		achabae sp. nov.	30	1.41	1.33	[3]
		bergstroensis sp. nov.	?	1.73	1.60	[21]
		bergstroensis	11	1.96	1.77	[51]
		boumendjelae sp. nov.	70	1.89	1.55	[30]
		contracta sp. nov.	12	1.99	1.29	[5]
		densimura sp. nov.	14	1.65	1.43	
		domfrontensis sp. nov.	20	1.87	1.45	[3]
		elongata	6	1.57	1.34	[5]
		laurentiana sp. nov.	18	2.06	1.53	[38]
		ontariensis comb. nov. emend.	25	2.76	1.76	[2]
		ontariensis	12	2.27	1.56	[5]
	Pogonochitina	*simplex* sp. nov.	3	/	/	[48]
		secunda	20	2.00	1.49	[3]
	Belonechitina	*robusta* sp. nov.	41	2.07	/	[62]
		robusta	?	2.03	1.41	[21]
			50	2.15	1.98	[23]
			14	2.08	2.40	[19]
			37	2.92	2.20	
			4	1.12	1.20	
		americana n. comb.	110	2.70	2.50	[65]
		americana	104	2.50	1.86	[19]
			14	1.91	1.64	

		aspera	11	2.13	2.36	[66]
		brittanica sp. nov.	273	2.78	2.00	[19]
			72	1.90	2.10	
			4	1.80	1.56	
		cactacea	?	1.42	1.26	[21]
			25	2.03	1.54	[2]
		capitata	?	2.70	1.93	[21]
			3	1.20	1.17	[19]
			2	1.06	1.20	
			7	1.94	1.56	
		cavei sp. nov.	15	1.65	1.81	[17]
		ceredigionensis sp. nov.	44	2.33	1.67	[67]
		gutta sp. nov.	?	1.20	1.27	[10]
		intonsa sp. nov.	37	1.65	1.45	[57]
		lauensis sp. nov.	?	1.26	1.25	[10]
		llangrannogensis sp. nov.	21	1.90	1.50	[67]
		martinica sp. nov.	20	1.37	1.40	[38]
		meifodensis sp. nov.	16	1.67	1.48	[17]
		micracantha	35	2.01	1.48	[23]
			62	2.23	1.60	[3]
			15	1.62	1.33	[24]
			159	3.60	2.20	[19]
			19	1.71	1.25	
			4	1.20	1.20	
			8	1.79	1.55	
		noraensis sp. nov.	170	2.73	2.05	[55]
		henryi sp. nov.	20	1.95	1.57	[3]
		hirsuta sp. nov.	?	1.23	1.09	[21]
		hirsuta	12	/	1.33	[23]
			10	1.27	1.29	[61]
			12	1.56	1.36	[65]
		oeselensis sp. nov.	18	1.39	1.60	[39]
		parvispinata sp. nov.	18	1.83	1.52	[38]
		postrobusta	69	2.48	2.89	[66]
		pseudarabiensis sp. nov.	229	2.00	1.91	
		pygmaea sp. nov.	15	1.39	1.35	[24]
		robardeti sp. nov.	25	2.93	1.74	[3]
		tribulosa sp. nov.	6	2.04	1.18	[23]

		tenuicomata sp. nov.	34	1.73	1.79	[4]
		tenuispinata sp. nov.	22	2.00	1.44	[38]
		visbyensis sp. nov.	?	1.30	1.37	[10]
		vulgaris	24	1.55	1.33	[19]
		wesenbergensis	40	1.91	1.82	[23]
			21	1.62	1.95	[19]
	Acanthochitina	*barbata* sp. nov.	/	/	/	[20]
		barbata	25	1.62	1.20	[6]
			?	1.81	1.48	[21]
			4	1.29	1.20	[61]
			34	1.56	1.74	[4]
		cancellata	8	1.44	1.25	[24]
		latebrosa sp. nov.	37	2.00	1.36	[19]
		merga	15	1.50	1.20	[24]
		pudica sp. nov.	6	1.47	1.50	[19]
		rashidi sp. nov.	25	1.50	1.31	[2]
	Hercochitina	*crickmayi* sp. nov.	?	1.96	/	[37]
		crickmayi	50	1.78	1.36	[23]
			20	1.96	1.44	[49]
		cristata sp. nov.	20	1.41	1.23	[24]
		downiei sp. nov.	21	1.39	1.42	[6]
		filamentosa sp. nov.	15	1.67	1.36	[49]
		frangiata sp. nov.	86	2.84	1.89	[19]
		grandispina sp. nov.	6	1.79	1.40	[49]
		lindsayensis	8	1.14	1.14	[24]
		lineola	5	1.35	1.29	
		minuta sp. nov.	10	1.69	1.25	[49]
		modesta sp. nov.	6	1.26	1.08	[7]
		multiansata sp. nov.	12	1.29	1.22	[4]
		normalis sp. nov.	20	2.00	1.50	[49]
		pinguis	10	1.32	1.30	[24]
		seriespinosa sp. nov.	50	1.61	1.48	[23]
		seriespinosa	6	1.33	1.40	[63]
		spinetum	3	1.48	1.30	[65]
		turnbulli sp. nov.	35	1.33	1.46	[23]
		violana sp. nov.	331	3.37	2.18	This study
	Spinachitina	*cervicornis* sp. nov.	3	1.22	1.16	[20]
		cervicornis	?	1.55	1.20	[21]
			16	1.75	1.45	[19]
		bulmani comb. nov.	18	1.17	1.28	[6]
		bulmani	8	1.93	1.22	[49]
			10	1.38	1.33	[7]

			20	1.86	1.18	[24]
			114	2.36	1.82	[65]
			6	1.43	1.36	[19]
			16	2.08	1.55	
		coronata?	18	1.87	1.50	
			14	1.83	1.40	
			6	1.38	1.20	
			2	1.06	1.22	
			13	1.91	1.51	
		geerti sp. nov.	15	2.28	1.88	[15]
		fragilis	26	2.15	1.45	[66]
			72	2.18	1.46	[50]
		fossensis sp. nov.	85	2.14	1.67	[51]
		fossensis	142	2.33	2.00	[19]
		katherinae sp. nov.	85	2.60	2.10	
		multiradiata	?	1.48	1.57	[21]
			88	2.38	1.90	[19]
			30	1.68	1.60	
		oulebsiri sp. nov.	43	1.70	1.53	[68]
		oulebsiri	46	2.57	1.82	[69]
		penbryniensis sp. nov.	94	4.07	2.22	[67]
		suecica sp. nov.	?	2.22	1.40	[21]
		taugourdeaui	10	2.36	2.20	[19]
		verniersi sp. nov.	25	2.42	1.50	[69]
Lagenochitinidae	*Alhajrichitina*	*adamantea* sp. nov.	25	1.64	1.40	[5]
	Saharochitina	*jaglini* sp. nov.	48	1.98	1.40	[30]
		fungiformis	6	1.11	1.17	[19]
			34	2.14	1.70	
			13	1.63	1.27	
			47	2.00	1.60	
			21	1.58	1.27	
			20	2.00	1.60	
		lepta sp. nov.	25	1.45	1.22	[2]
		lepta	34	2.13	1.82	[19]
	Sphaerochitina	*sphaerocephala* sp. nov.	2	1.06	1.01	[70]
		gracqui	6	1.45	1.29	[24]
		lycoperdoides	40	1.56	1.35	[3]
		patula sp. nov.	23	1.47	1.50	[8]
	Lagenochiti	*baltica* sp. nov.	?	1.25	/	[20]

	na	*baltica*	18	1.51	1.52	[6]
			?	2.07	1.78	[21]
			10	1.88	1.64	[61]
			23	2.35	2.50	[19]
			24	1.52	1.42	
			13	1.72	2.06	
			16	1.59	1.21	[44]
		avelinoi	3	1.19	1.07	[12]
		combazi	50	2.92	2.67	[55]
		capax sp. nov.	2	1.71	1.89	[6]
		compactilis sp. nov.	20	3.26	1.29	[23]
		cylindrica	10	1.60	1.17	[6]
		dalbyensis sp. nov.	?	1.50	1.60	[21]
		dalbyensis	67	2.00	1.50	[40]
			30	1.74	1.65	[3]
		destombesi	76	1.41	1.27	[44]
		esthonica	3	1.86	1.13	[6]
			more than 6	1.69	1.57	[47]
			4	1.46	1.41	[3]
			10	1.29	1.46	[46]
			3	1.32	1.37	[44]
		deunffi	60	1.92	1.66	[3]
		navicula	30	1.38	1.24	
		obeligis sp. nov.	60	2.17	2.11	
		obeligis	25	1.93	1.64	[44]
		pestovoensis	24	2.28	2.15	[71]
		pirum sp. nov.	20	1.77	1.25	[53]
		pirum	8	1.27	1.43	[54]
			159	2.58	2.65	[63]
		prussica	?	1.66	2.00	[21]
			18	1.59	1.68	[19]
			3	1.50	2.00	
			2	1.45	1.40	
		shelvensis sp. nov.	3	1.25	1.29	[6]
	Anthochitina	*superba* sp. nov.	30	1.60	/	[72]
		jawfensis sp. nov.	15	1.29	/	[33]
	Sagenachitina	*oblonga* sp. nov.	3	1.44	1.45	[73]
		oblonga	4	2.31	1.11	[3]
			5	1.50	1.12	
	Cyathochitina	*campanulaeformis* sp. nov.	?	1.20	/	[20]

		campanulaeformis	?	2.14	/	[74]
			45	1.68	1.69	[6]
			?	2.13	/	[21]
			20	1.89	1.81	[3]
			36	1.85	1.74	
			8	1.95	1.38	[24]
			97	2.09	3.29	[19]
			2	1.33	1.45	
			15	1.69	2.00	
			3	1.79	1.67	
			26	2.50	2.77	
			259	2.41	3.52	[66]
			32	2.07	2.16	[50]
			1256	3.77	3.87	[63]
		angusta sp. nov.	?	1.55	1.27	[29]
		calix	6	1.21	1.19	[6]
			9	1.81	1.48	[3]
			10	2.19	2.40	
			4	1.27	1.45	[19]
		giraffe sp. nov. =*cycnea* sp. nov.	89	2.23	1.90	[63]
		kuckersiana	35	2.05	1.61	[6]
			?	1.38	1.41	[21]
			15	1.58	1.54	[49]
			10	1.45	1.18	[7]
			20	1.65	1.89	[24]
			43	2.23	2.29	[19]
			14	2.08	2.10	
			14	2.07	2.80	
			2	1.40	1.25	
			18	1.65	1.86	[66]
		latipatagium	11	2.17	1.69	[19]
		macastyensis sp. nov.	12	1.59	1.29	[7]
		megacalix sp. nov.	20	1.69	1.71	[41]
		neolatipatagium sp. nov.	40	2.13	2.03	[15]
		protocalix sp. nov.	42	2.11	2.05	[3]
			20	2.50	1.78	
		raricostata sp. nov.	3	1.26	1.32	[41]
		touggourtensis sp. nov.	80	2.11	1.79	[30]

		vaurealensis sp. nov.	20	1.53	1.44	[61]
		vaurealensis	5	1.80	1.40	[49]
			4	1.62	1.20	[7]
		varennensis sp. nov.	20	1.38	1.43	[3]
	Parisochitina	*perforata* sp. nov.	4	1.20	1.37	[75]
	Pellichitina	*pellucida* sp. nov.	2	/	/	[73]
		pellucida	11	1.45	1.70	[26]
	Urochitina	*simplex* sp. nov.	1	/	/	[13]
		bastosi	3	1.18	1.26	[12]
	Fungochitina	*funiformis* sp. nov.	1	/	/	[20]
		actonica sp. nov.	6	1.24	1.20	[6]
		actonica	71	1.86	1.70	[19]
		illinoisensis sp. nov.	29	1.41	1.34	[50]
		impia sp. nov.	?	1.31	1.20	[10]
		kosovensis sp. nov.	50	1.73	1.82	[9]
		kosovensis	5	1.72	1.25	[32]
		merrelli sp. nov.	13	1.46	1.24	[15]
		pilosa	40	1.64	1.59	[12]
		spinifera	14	2.00	1.61	[19]
	Angochitina	*echinata* sp. nov.	2	1.14	/	[20]
		echinata	?	1.33	1.50	[10]
		capillata	36	1.74	1.46	[23]
		caeciliae	20	1.20	1.28	[3]
		carvalhoi sp. nov.	12	1.77	1.92	[12]
		comosa	20	1.45	1.43	[3]
		communis sp. nov.	25	1.45	1.21	[6]
		communis	132	1.81	2.83	[65]
			70	1.86	1.80	[19]
			34	1.73	1.69	
		concava sp. nov.	?	1.25	1.25	[10]
		curvata sp. nov.	15	1.27	1.13	[29]
		dicramum sp. nov.	12	1.56	1.49	[6]
		hansonica sp. nov.	20	1.41	1.42	[38]
		hansonica	5	1.22	1.09	[50]
		katzeri sp. nov.	41	1.43	2.00	[12]
		lycoperdoides sp. nov.	?	1.32	1.18	[10]
		loboziaki sp. nov.	28	1.95	1.67	[12]
		longicollis	42	2.22	1.53	[17]
		mourai	95	2.14	2.17	[12]
		muscosa sp. nov.	30	1.75	1.43	[3]

		punctata sp. nov.	50	1.77	1.60	
		thadeui sp. nov.	40	1.55	1.30	
	Muscochitina	*olivieri* sp. nov.	37	1.75	1.75	[15]
	Ramochitina	*ramosi* sp. nov.	1	/	/	[76]
		clarkei sp.nov.	14	1.34	1.33	[12]
		corniculata sp. nov.	?	1.24	1.44	[10]
		cornuta sp. nov.	?	1.22	1.33	
		derbyi sp. nov.	46	2.50	1.54	[12]
		famennense sp. nov.	8	1.35	1.22	
		goliath sp. nov.	16	1.39	1.46	[3]
		hartti sp. nov.	13	1.47	1.30	[12]
		martinssoni sp. nov.	?	1.11	1.08	[10]
		militaris sp. nov.	?	1.11	1.17	
		oliveirai sp. nov.	50	1.46	1.46	[12]
		praeritae sp. nov.	6	1.32	1.29	
		racheboeufi sp. nov.	12	1.39	1.26	[3]
		ritae sp. nov.	73	1.90	1.67	[12]
		spinipes	?	1.14	1.15	[10]
		spinosa	?	1.32	1.33	
		tabernaculifera sp. nov.	?	1.29	1.30	
		uncinata sp. nov.	?	1.23	1.40	
		valbyttiensis sp. nov.	?	1.22	1.23	
		villosa sp. nov.	?	1.12	1.36	
	Ancyrochitina	*ancyrea* sp. nov.	3	1.13	1.08	[20]
		ancyrea	52	2.11	1.78	[65]
		ancyrea	32	1.60	1.55	[66]
		ancyrea	10	1.35	1.30	[50]
		ansarviensis sp. nov.	?	1.33	1.30	[10]
		alaticornis sp. nov.	35	2.17	1.89	[6]
		alhajrii sp. nov.	37	1.59	1.39	[15]
		asterigis sp. nov.	30	1.50	1.42	[3]
		brevis	40	1.75	1.82	[32]
		camilleae sp. nov.	30	1.50	1.47	[15]
		corniculans sp. nov.	25	2.26	1.53	[23]
		desmea	?	1.35	1.17	[10]
		ellisbayensis sp. nov.	14	1.21	1.44	[52]
		floris sp. nov.	4	1.35	1.30	[8]
		fragilis	50	2.39	1.40	[3]
		gutnica sp. nov.	?	1.23	1.33	[10]

		laevaensis	4	1.30	1.21	[66]
		libyensis sp. nov.	43	2.31	1.90	[8]
		merga sp. nov.	25	1.55	1.40	[2]
		merga	20	1.30	1.29	[61]
		merga	25	1.75	1.50	[19]
		merga	1	/	/	[5]
		morzadeci sp. nov.	5	1.15	1.31	[3]
		mullinsi sp. nov.	?	1.64	1.42	[39]
		onniensis sp. nov.	25	1.49	1.21	[6]
		onniensis	82	1.87	1.80	[19]
		pachyderma sp. nov.	?	1.19	1.14	[10]
		parafragilis sp. nov.	15	1.38	1.10	[33]
		pedavis sp. nov.	?	1.52	1.35	[10]
		regularis	40	1.68	1.50	[32]
		spongiosa	20	1.73	1.33	[49]
		spongiosa	10	1.33	1.13	[7]
		spongiosa	10	1.37	1.29	[24]
		tomentosa	25	1.49	1.36	[3]
	Clathrochitina	*clathrata* sp. nov.	1	/	/	[62]
		clathrata	?	/	/	[10]
		perexilis sp. nov.	11	/	/	[52]
		postconcinna sp. nov.	12	/	/	
		sylvanica sp. nov.	25	1.45	1.37	[2]
	Plectochitina	*carminae* sp. nov.	?	1.60	/	[31]
		alisawyiahensis sp. nov.	30	1.68	1.57	[15]
		alnaimi sp. nov.	55	1.81	1.71	
		jacquelineae sp. nov.	40	2.35	1.74	
		lucasi sp. nov.	36	1.96	1.42	
		nodifera	28	1.22	1.20	[66]
	Alpenachitina	*eisenacki* sp. nov.	11	1.32	1.44	[77]
	Sommerochitina	*langei* sp. nov.	106	2.87	/	[78]

Electronic supplementary material §3. Egg size variation in extant invertebrate

A literature survey was conducted to obtain estimates of the CV of egg size of aquatic (mostly marine) invertebrates for comparison to our estimate of the CV in *H. violana* size. We attempted to obtain estimates from a broad range of species representing phylogenetic diversity at the Phylum, Class and Order levels.

§3.1. Criteria of CV data selecting

We accepted reported CV values or we computed CV from means and standard deviations as reported in those papers. In cases where multiple estimates existed for the same species from different workers or localities, we accepted both estimates. When a study reported multiple estimates of egg size and its variation (from multiple samples or experiments conducted in the same study) we computed a mean CV for all reported values within that study. In cases in which standard error (SE) rather than SD was reported and for which sample size (n) was reported, we converted SE to SD (SD $= \sqrt{n} * SE$) to calculate CV. Only CV estimates based upon among-clutch (among-female) variation were used, because these are the appropriate level of comparison needed to characterize egg size variance within a species [79]. Many reports of CV of egg size within a single clutch were therefore not included in our dataset. We rejected studies in which CV or SD is based on fewer than five females, because between-female variation in egg size is common [79] and estimates based on a very small number of individual clutches would inflate estimates of variance. Even samples larger than this are likely to exhibit higher variance estimates than larger

samples, but we chose to err on the side of having more phylogenetic representation in the dataset. Thus some of the CV estimates we obtained would likely have been even smaller if larger numbers of clutches were used to characterize the species variability in egg size. It is important to note that had we imposed an even more stringent criterion with respect to sample size (numbers of individuals used to characterize egg size variance) the mean CV would have been even smaller than that which we report here.

We characterized the dataset of CV values statistically, computing the mean, median and standard error of the distribution of CV values. We then assessed whether the distribution was normal, and therefore appropriate for applying normative statistics to test the hypothesis that our observation of variability in *B. violana* was likely to belong to the same distribution. To do this we used the one sample t-test [80].

Data presented below are arranged by Phylum in approximate phylogenetic order (basal lineages first) and then alphabetically with respect Class, Order, Genus and species. Species and Order names follow current taxonomic allocations as updated in the World Registry of Marine Species (http://www.marinespecies.org). Taxon names from the original reports that have been superseded are shown in parentheses.

§3.2. A survey of CV of egg size (or neonate size) among aquatic, mostly marine invertebrates across six phyla.

Phylum: Class: Order	Species	Habitat	Trait	CV	Reference

				(%)	
Bryozoa: Gymnolaemata: Cheilostomatida	*Bugula neritina*	marine	Egg diameter	6.9	[81]
Annellida: Polychaeta: Sabellida	*Galeolaria caespitosa*	marine	Egg diameter	1.99	[81]
Mollusca: Bivalvia: Cardida	*Limecola* (*Macoma*) *balthica*	marine	Egg diameter	6.50	[82]
Mollusca: Gastropoda: Lepetellida	*Diadora aspersa*	marine	Egg diameter	4.9	[81]
Mollusca: Gastropoda: Lepetellida	*Haliotis rubra*	marine	Egg diameter	7.35	[83]
Mollusca: Gastropoda: Littorinimorpha	*Crepidula atrasolea*	marine	Egg diameter	4.98[c]	[84]
Mollusca: Gastropoda: Littorinimorpha	*Crepidula ustulatulina*	marine	Egg diameter	6.34[c]	[84]
Mollusca: Gastropoda: Patellogastropoda[a]	*Lottia pelta*	marine	Egg diameter	2.4	[81]
Mollusca: Opistobranchia: Heterobranchia[a]	*Alderia modesta*	marine	Egg volume	11.75	[81]
Mollusca: Opistobranchia: Heterobranchia[a]	*Alderia modesta*	marine	Egg volume	12.37	[81]
Arthropoda: Crustacea: Diplostraca	*Daphnia galeata mendotae*	freshwater	Neonate mass	4.499	[85]
Arthropoda: Crustacea: Diplostraca	*Daphnia parvula*	freshwater	Neonate mass	4.999	[85]
Arthropoda: Crustacea: Diplostraca	*Daphnia pullicaria*	freshwater	Neonate mass	3.437	[85]
Arthropoda: Crustacea: Calanoida	*Acartia tonsa*	marine	Egg diameter	1.26	[86]
Arthropoda: Crustacea: Calanoida	*Calanus finmarchicus*	marine	Egg diameter	5.30[b]	[87]
Arthropoda:	*Calanus finmarchicus*	marine	Egg	2.32	[88]

Crustacea: Calanoida			diameter		
Arthropoda: Crustacea: Calanoida	*Calanus glacialis*	marine	Egg diameter	1.79	[88]
Arthropoda: Crustacea: Calanoida	*Calanus marshallae*	marine	Egg diameter	3.09	[88]
Arthropoda: Crustacea: Calanoida	*Calanus pacificus*	marine	Egg diameter	2.24	[88]
Arthropoda: Crustacea: Calanoida	*Neocalanus tonsus*	marine	Egg diameter	0.70	[89]
Arthropoda: Crustacea: Decapoda	*Panulirus marginatus*	marine	Egg diameter	6.42	[90]
Arthropoda: Crustacea: Decapoda	*Scyllarides squammosus*	marine	Egg diameter	0.895	[91]
Echinodermata: Asteroidea: Forcipulatida	*Asterias forbesi*	marine	Egg volume	16.31	[81]
Echinodermata: Asteroidea: Forcipulatida	*Uniophora granifera*	marine	Egg diameter	6.3	[81]
Echinodermata: Asteroidea: Paxillosida	*Luidia clathrata*	marine	Egg volume	8.52	[81]
Echinodermata: Asteroidea: Spinulosida	*Echinaster modestus*	marine	Egg diameter	16.21	[81]
Echinodermata: Asteroidea: Spinulosida	*Echinaster modestus*	marine	Egg diameter	8.27	[81]
Echindermata: Asteroidea: Spinulosida	*Henricia sp.*	marine	Egg diameter	11.88	[92]
Echindermata: Asteroidea: Valvatida	*Mediaster aequalis*	marine	Egg diameter	4.35	[92]
Echinodermata: Asteroidea: Valvatida	*Meridiastra calcar*	marine	Egg diameter	3.87	[81]
Echinodermata: Asteroidea: Valvatida	*Meridiastra gunnii*	marine	Egg diameter	5.07	[81]
Echinodermata: Asteroidea: Valvatida	*Meridiastra occidens*	marine	Egg diameter	4.42	[81]
Echinodermata: Asteroidea: Valvatida	*Parvulastra parvivipara*	marine	Egg diameter	7.16	[81]
Echindermata: Asteroidea: Valvatida	*Solaster dawsoni*	marine	Egg diameter	5.40	[92]

Echindermata: Asteroidea: Valvatida	*Solaster endeca*	marine	Egg diameter	10.34	[92]
Echinodermata: Asteroidea: Valvatida	*Solaster simpsoni*	marine	Egg volume	9.1	[93]
Echinodermata: Asteroidea: Velatida	*Pteraster tesselatus*	marine	Egg volume	5.8	[94]
Echindermata: Asteroidea: Velatida	*Pteraster tesselatus*	marine	Egg diameter	16.46	[92]
Echinodermata: Echinoidea: Camarodonta	*Lytechinus variegatus*	marine	Egg volume	9.05	[81]
Echinodermata: Echinoidea: Camarodonta	*Strongylocentrotus droebachiensis*	marine	Egg volume	4.96	[81]
Echinodermata: Echinoidea: Camarodonta	*Strongylocentrotus droebachiensis*	marine	Egg diameter	2.097	[95]
Echinodermata: Echinoidea: Clypeasteroida	*Clypeaster rosaceus*	marine	Egg diameter	2.74	[81]
Echinodermata: Echinoidea: Clypeasteroida	*Dendraster exentricus*	marine	Egg diameter	3.5	[81]
Echinodermata: Echinoidea: Clypeasteroida	*Encope* (*aberrans*) *michelini*	marine	Egg volume	11.17	[81]
Echinodermata: Holothuroidea: Dendrochirotida	*Cucumaria miniata*	marine	Egg diameter	16.67	[92]
Echinodermata: Holothuroidea: Dendrochirotida	*Psolus chitonoides* (*chitinoides*)	marine	Egg diameter	10.00	[92]
Chordata: Ascidia: Phlebobranchia	*Ciona intestinalis*	marine	Egg diameter	5.17	[81]
Chordata: Ascidia: Stolidobranchia	*Pyura fissa*	marine	Egg diameter	5.21	[81]
Chordata: Ascidia: Stolidobranchia	*Pyura stolonifera*	marine	Egg diameter	9.18	[81]
Chordata: Ascidia: Stolidobranchia	*Styela plicata*	marine	Egg diameter	7.90	[81]

66 **Superscript Caption**

[a] Subclass, not order.

[b] Computed from data (mean and standard deviation) in Table 4; We report the mean CV of five reported values.

[c] Computed from data (mean and standard deviation) in Table 1; We report the mean CV of two estimates for each species.

References

[1] Wilson, L. & Hedlund, R. 1964 Calpichitina scabiosa, a new chitinozoan from the Sylvan Shale (Ordovician) of Oklahoma. *Oklahoma Geological Notes* **24**, 161-164.

[2] Jenkins, W.A.M. 1970 Chitinozoa from the Ordovician Sylvan shale of the Arbuckle mountains, Oklahoma. *Palaeontology* **13**, 261-288.

[3] Paris, F. 1981 *Les Chitinozoaires dans le Paléozoïque de sud-ouest de l'Europe: cadre géologique, étude systématique, biostratigraphie*, Mémoires de la Société Géologique et Minéralogique de Bretagne.

[4] Paris, F., Verniers, J., Miller, M.A., Melvin, J. & Wellman, C.H. 2015 Late Ordovician–earliest Silurian chitinozoans from the Qusaiba-1 core hole (North Central Saudi Arabia) and their relation to the Hirnantian glaciation. *Review of Palaeobotany and Palynology* **212**, 60-84.

[5] Al-Shawareb, A., Miller, M. & Vecoli, M. 2017 Late Ordovician (Katian) chitinozoans from northwest Saudi Arabia: Biostratigraphic and paleoenvironmental implications. *Revue de Micropaléontologie* **60**, 333-369.

[6] Jenkins, W.A.M. 1967 Ordovician chitinozoa from Shropshire. *Palaeontology* **10**, 436-488.

[7] Achab, A. 1978 Sur quelques chitinozoaires de la formation de vauréal et de la formation de macasty (Ordovicien supérieur), ile d'anticosti, Québec, Canada. *Review of Palaeobotany and Palynology* **25**, 295-314.

[8] Jaglin, J. 1986 Nouvelles espèces de Chitinozoaires du Pridolí de Libye. *Revue de Micropaléontologie* **29**, 44-54.

[9] Paris, F. & Kříž, J. 1984 Nouvelles espéces de Chitinozoaires à la limite Ludlow/Pridoli en Tchécoslovaquie. *Review of Palaeobotany and Palynology* **43**, 155-177.

[10] Laufeld, S. 1974 Silurian chitinozoa from Gotland. *Fossils and Strata* **5**, 1-130.

[11] Staplin, F.L. 1961 Reef-controlled distribution of Devonian microplankton in Alberta. *Palaeontology* **4**, 392-424.

[12] Grahn, Y. & de Melo, J.H.G. 2002 Chitinozoan biostratigraphy of the Late Devonian formations in well Caima PH-2, Tapajós River area, Amazonas Basin, northern Brazil. *Review of Palaeobotany and Palynology* **118**, 115-139.

[13] Taugourdeau, P. & de Jekhowsky, B. 1960 *Répartition et description des chitinozoaires siluro-dévoniens de quelques sondages de la CREPS, de la CFPA et de la SN Repal au Sahara*, Revue de l'Institut Francais du Pétrole.

[14] Taugourdeau, P. 1967 Néotypes de chitinozoaires. *Revue de micropaléontologie* **9**, 258-264.

[15] Paris, F., Miller, M.A. & Zalasiewicz, J. 2015 Early Silurian chitinozoans from the Qusaiba type area, north Central Saudi Arabia. *Review of Palaeobotany and Palynology* **212**, 127-186.

[16] Soufiane, A. & Achab, A. 2000 Chitinozoan zonation of the Late Ordovician and the Early Silurian of the island of Anticosti, Québec, Canada. *Review of Palaeobotany and Palynology* **109**, 85-111.

[17] Mullins, G.L. & Loydell, D.K. 2001 Integrated Silurian chitinozoan and graptolite biostratigraphy of the Banwy River section, Wales. *Palaeontology* **44**, 731-781.

[18] Vandenbroucke, T.R.A., Rickards, B. & Verniers, J. 2005 Upper Ordovician chitinozoan biostratigraphy from the type Ashgill area (Cautley district) and the Pus Gill section (Dufton district, Cross Fell Inlier), Cumbria, northern England. *Geological Magazine* **142**, 783-807.

[19] Vandenbroucke, T.R.A. 2008 Upper Ordovician chitinozoans from the British historical type areas and adjacent key sections. *Monograph of the Palaeontographical Society, London* **161**, 1-113.

[20] Eisenack, A. 1931 Neue Mikrofossilien des baltischen Silurs. I. *Paläontologische Zeitschrift* **13**, 74–118.

[21] Laufeld, S. 1967 Caradocian Chitinozoa from Dalarna, Sweden. *Geologiska Föreningen i Stockholm Förhandlingar* **89**, 275-349.

[22] Schallreuter, R. 1981 Chitinozoen aus dem Sularpschiefer (Mittelordoviz) von Schonen (Schweden). *Palaeontographica Abteilung B* **178**, 89-142.

[23] Jenkins, W.A.M. 1969 *Chitinozoa from the Ordovician Viola and Fernrale limestones of the Arbuckle Mountains, Oklahoma*, Palaeontological Association.

[24] Achab, A. 1987 Chitinozoaires du Caradoc supérieur–Ashgill inférieur du Québec, Canada. *Canadian Journal of Earth Sciences* **24**, 1212-1234.

[25] Poumot, C. 1968 Amphorachitina, Ollachitina, Velatachitina, trois nouveaux genres de chitinozoaires de l'Erg Oriental (Algérie-Tunesie). *Bulletin du Centre de Recherche de Pau* **2**, 45-55.

[26] Achab, A., Asselin, E. & Soufiane, A. 1993 New morphological characters observed in the order Operculatifera and their implications for the suprageneric chitinozoan classification. *Palynology* **17**, 1-9.

[27] Eisenack, A. 1937 Neue Mikrofossilien des baltischen Silurs. IV. *Paläontologische Zeitschrift* **19**, 217-243.

[28] Paris, F. 1976 Les chitinozoaires. *Les schistes et calcaires éodévoniens de Saint-Cénéré (Massif armoricain, France). Sédimentologie, Paléontologie, Stratigraphie. Mémoires de la Société géologique et minéralogique de Bretagne* **19**, 93-133.

[29] Nõlvak, J. & Grahn, Y. 1993 Ordovician chitinozoan zones from Baltoscandia. *Review of Palaeobotany and Palynology* **79**, 245–269.

[30] Oulebsir, L. & Paris, F. 1993 Nouvelles espèces de chitinozoaires dans l'Ordovicien inférieur et moyen du sud-est du Sahara algérien. *Revue de Micropaléontologie* **36**, 269-292.

[31] Cramer, F.H. 1964 Microplankton from three Palaeozoic formations in the province of Leon, NW-Spain. *Leidse Geologische Mededelingen* **30**, 253-361.

[32] Jaglin, J. & Paris, F. 2002 Biostratigraphy, biodiversity and palaeogeography of late Silurian chitinozoans from A1-61 Borehole (north–western Libya). *Review of Palaeobotany and Palynology* **118**, 335-358.

[33] Al-Hajri, S. & Paris, F. 1998 Age and palaeoenvironmentof the Sharawra Member (Silurian of North-Western Saudi Arabia). *Geobios* **31**, 3-12.

[34] Eisenack, A. 1934 Neue mikrofossilien des baltischen Silurs. III. und neue mikrofossilien des böhmischen Silurs. I. *Paläontologische Zeitschrift* **16**, 52-76.

[35] Grahn, Y. 2005 Silurian and Lower Devonian chitinozoan taxonomy and biostratigraphy of the Trombetas Group, Amazonas Basin, northern Brazil. *Bulletin of Geosciences* **80**, 245-276.

[36] Sutherland, S. 1994 *Ludlow chitinozoans from the type area and adjacent regions*, Palaeontographical Society.

[37] Jansonius, J. 1964 Morphology and classification of some Chitinozoa. *Bulletin of Canadian Petroleum Geology* **12**, 901-918.

[38] Soufiane, A. & Achab, A. 2000b Upper Ordovician and lower Silurian chitinozoans from central Nevada and Arctic Canada. *Review of Palaeobotany and Palynology* **113**, 165-187.

[39] Nestor, V. 2005 Chitinozoans of the *Margachitina margaritana* Biozone and the Llandovery--Wenlock boundary in West Estonian drill cores. *Proceedings of the Estonian Academy of Sciences, Geology* **54**, 87-111.

[40] Vandenbroucke, T.R.A. 2004 Chitinozoan biostratigraphy of the Upper Ordovician Fågelsång GSSP, Scania, southern Sweden. *Review of Palaeobotany and Palynology* **130**, 217-239.

[41] Liang, Y., Paris, F. & Tang, P. 2017 Middle–Late Ordovician chitinozoans from the Yichang area, South China. *Review of Palaeobotany and Palynology* **244**, 26-42.

[42] Eisenack, A. 1955 Neue Chitinozoen aus dem Silur des Baltikums und dem Devon der Eifel. *Senckenbergiana lethaea* **36**, 311-319.

[43] Grahn, Y. 1981 *Middle Ordovician Chitinozoa from Öland*, Liber Kartor [in Komm.].

[44] Wang, W., Feng, H., Vandenbroucke, T.R.A., Li, L. & Verniers, J. 2013 Chitinozoans from the Tremadocian graptolite shales of the Jiangnan Slope in South China. *Review of palaeobotany and palynology* **198**, 45-61.

[45] de la Puente, G.S. & Rubinstein, C.V. 2009 Late Tremadocian chitinozoans and acritarchs from northwestern Argentina (Western Gondwana). *Review of Palaeobotany and Palynology* **154**, 65-78.

[46] Achab, A. 1986 Assemblages de chitinozoaires dans l'Ordovicien inférieur de l'est du Canada. *Canadian Journal of Earth Sciences* **23**, 682-695.

[47] Achab, A. 1980 Chitinozoaires de l'Arenig inférieur de la Formation de Lévis (Québec, Canada). *Review of Palaeobotany and Palynology* **31**, 219-239.

[48] Taugourdeau, P. 1961 Chitinozoaires du Silurien d'Aquitaine. *Revue de micropaléontologie* **6**, 135-154.

[49] Achab, A. 1977b Les chitinozoaires de la zone à Climacograptus prominens elongatus de la Formation de Vauréal (Ordovicien supérieur), Ile d'Anticosti, Québec. *Canadian Journal of Earth Sciences* **14**, 2193-2212.

[50] Butcher, A., Mikulic, D.G. & Kluessendorf, J. 2010 Late Ordovician–Early Silurian chitinozoans

from north-eastern and western Illinois, USA. *Review of Palaeobotany and Palynology* **159**, 81-93.

[51] Vanmeirhaeghe, J. & Verniers, J. 2004 Chitinozoan bio-and lithostratigraphical study of the Ashgill Fosses and Génicot Formations (Condroz Inlier, Belgium). *Review of Palaeobotany and Palynology* **130**, 241-267.

[52] Soufiane, A. & Achab, A. 2000a Chitinozoan zonation of the Late Ordovician and the Early Silurian of the island of Anticosti, Québec, Canada. *Review of Palaeobotany and Palynology* **109**, 85-111.

[53] Achab, A. 1982 Chitinozoaires de l'Arenig supérieur (Zone D) de la Formation de Lévis, Québec, Canada. *Canadian Journal of Earth Sciences* **19**, 1295-1307.

[54] Achab, A. 1983 Chitinozoaires du Llanvirn (formation de Table Head) de la partie occidentale de Terre-Neuve, Canada. *Canadian Journal of Earth Sciences* **20**, 918-931.

[55] Playford, G. & Miller, M.A. 1988 Chitinozoa from lower Ordovician strata of the Georgina basin, Queensland (Australia). *Geobios* **21**, 17-39.

[56] Nõlvak, J. 2007 A new chitinozoan species from the Upper Ordovician of the East Baltic. *Estonian Journal of Earth Sciences* **56**.

[57] Nõlvak, J. & Bauert, G. 2015 New biostratigraphically important chitinozoans from the Kukruse Regional Stage, Upper Ordovician of Baltoscandia. *Estonian Journal of Earth Sciences* **64**, 218.

[58] Grahn, Y. & Nõlvak, J. 2007 Ordovician Chitinozoa and biostratigraphy from Skåne and Bornholm, southernmost Scandinavia—an overview and update. *Bulletin of Geosciences* **82**, 11-26.

[59] Eisenack, A. 1968 Über Chitinozoen des baltischen Gebietes. *Palaeontographica Abteilung A*, 137-198.

[60] Eisenack, A. 1939 Chitinozoen und Hystrichosphaerideen im Ordovizium des Rheinischen

Schiefergebirges. *Senchenbergiana* **21**, 135–153.

[61] Achab, A. 1977a Les chitinozoaires de la zone à Dicellograptus complanatus Formation de Vauréal, Ordovicien supérieur, Ile d'Anticosti, Québec. *Canadian Journal of Earth Sciences* **14**, 413-425.

[62] Eisenack, A. 1959 Neotypen baltischer Silur-Chitinozoen und neue Arten. *Neues Jahrbuch für Geologie und Paläontologie, Abhandlungen* **108**, 1-20.

[63] Hennissen, J., Vandenbroucke, T.R.A., Chen, X., Tang, P. & Verniers, J. 2010 The Dawangou auxiliary GSSP (Xinjiang autonomous region, China) of the base of the Upper Ordovician Series: putting global chitinozoan biostratigraphy to the test. (Geological Society of London.

[64] Nõlvak, J. 2012 A new chitinozoan species from the Middle Ordovician of Estonia. *Estonian Journal of Earth Sciences* **61**, 120.

[65] Vandenbroucke, T.R.A., Verniers, J. & Clarkson, E.N. 2002 A chitinozoan biostratigraphy of the Upper Ordovician and lower Silurian strata of the Girvan area, Midland Valley, Scotland. *Earth and Environmental Science Transactions of The Royal Society of Edinburgh* **93**, 111-134.

[66] Butcher, A. 2009 Early Llandovery chitinozoans from Jordan. *Palaeontology* **52**, 593-629.

[67] Challands, T.J., Vandenbroucke, T.R.A., Armstrong, H.A. & Davies, J.R. 2014 Chitinozoan biozonation in the upper Katian and Hirnantian of the Welsh Basin, UK. *Review of Palaeobotany and Palynology* **210**, 1-21.

[68] Paris, F., Bourahrouh, A. & Hérissé, A. 2000 The effects of the final stages of the Late Ordovician glaciation on marine palynomorphs (chitinozoans, acritarchs, leiospheres) in well Nl-2 (NE Algerian Sahara). *Review of Palaeobotany and Palynology* **113**, 87-104.

[69] Vandenbroucke, T.R.A., Gabbott, S.E., Paris, F., Aldridge, R.J. & Theron, J.N. 2009 Chitinozoans and the age of the Soom Shale, an Ordovician black shale Lagerstätte, South Africa. *Journal of*

Micropalaeontology **28**, 53-66.

[70] Eisenack, A. 1932 Neue mikrofossilien des baltischen Silurs. II. *Paläontologische Zeitschrift* **14**, 257–277.

[71] Liang, Y., Servais, T., Tang, P., Liu, J. & Wang, W. 2017 Tremadocian (Early Ordovician) chitinozoan biostratigraphy of South China: An update. *Review of Palaeobotany and Palynology* **247**, 149-163.

[72] Eisenack, A. 1971 Weitere Mikrofossilien aus dem Beyrichienkalk (Silur). *Neues Jahrbuch für Geologie und Paläontologie, Monatshefte* **8**, 449-460.

[73] Benoît, A. & Taugourdeau, P. 1961 Sur quelques chitinozoaires de l'Ordovicien du Sahara. *Revue de I'Institut Francais du Pétrole* **16**, 1403–1421.

[74] Eisenack, A. 1962 Neotypen baltischer Silur-Chitinozoen und neue Arten. *Neues Jahrbuch für Geologie und Paläontologie, Abhandlungen* **114**, 291-316.

[75] Boumendjel, K. 1985 Nouvelles espèces de Chitinozoaires dans le Silurien et le Dévonien du Bassin d'Illizi (SE du Sahara algérien). *Revue de micropaléontologie* **28**, 155-166.

[76] Sommer, F. & Van Boekel, N. 1964 Quitinozoarios do Devoniano de Goiás. *Anais da Academia Brasileira de Ciências* **36**, 423-431.

[77] Dunn, D. & Miller, T. 1964 A distinctive chitinozoan from the Alpena Limestone (Middle Devonian) of Michigan. *Journal of Paleontology*, 725-728.

[78] Cruz, N.d.C. & Quadros, L. 1985 Sommerochitina langei, um novo fóssil-guia do Devoniano Superior da bacia do Parnaíba. *Coletânea de Trabalhos Paleontológicos, Ser. Geologia* **27**, 289-293.

[79] Bernardo, J. 1996 Maternal effects in animal ecology. *American Zoologist* **36**, 83-105.

[80] Sokal, R. & Rohlf, F. 1995 Biometry: the principles and practice of statistics in biological sciences.

WH Free Company, New York, USA.

[81] Marshall, D.J., Bonduriansky, R. & Bussière, L.F. 2008 Offspring size variation within broods as a bet-hedging strategy in unpredictable environments. *Ecology* **89**, 2506-2517. (doi:doi:10.1890/07-0267.1).

[82] Luttikhuizen, P.C., Honkoop, P.J.C. & Drent, J. 2011 Intraspecific egg size variation and sperm limitation in the broadcast spawning bivalve *Macoma balthica*. *Journal of Experimental Marine Biology and Ecology* **396**, 156-161. (doi:https://doi.org/10.1016/j.jembe.2010.10.017).

[83] Huchette, S., Soulard, J., Koh, C. & Day, R. 2004 Maternal variability in the blacklip abalone, Haliotis rubra leach (Mollusca: Gastropoda): effect of egg size on fertilisation success. *Aquaculture* **231**, 181-195.

[84] Collin, R. 2010 Repeatability of egg size in two marine gastropods brood order and female size do not contribute to intraspecific variation. *Marine Ecology Progress Series* **410**, 89-96.

[85] Tessier, A.J. & Consolatti, N.L. 1989 Variation in offspring size in *Daphnia* and consequences for individual fitness. *Oikos* **56**, 269-276. (doi:10.2307/3565347).

[86] Kleppel, G.S. 1992 Environmental regulation of feeding and egg production by *Acartia tonsa* off southern California. *Marine Biology* **112**, 57-65. (doi:10.1007/bf00349728).

[87] Rey, C., Carlotti, F., xe, ois, Tande, K. & Hygum, B.H. 1999 Egg and faecal pellet production of *Calanus finmarchicus* females from controlled mesocosms and in situ populations: influence of age and feeding history. *Marine Ecology Progress Series* **188**, 133-148.

[88] McLaren, I.A., Sevigny, J.M. & Corkett, C.J. 1988 Body sizes, development rates, and genome sizes among *Calanus* species. *Hydrobiologia* **167**, 275-284. (doi:10.1007/bf00026315).

[89] Ohman, M.D. 1987 Energy sources for recruitment of the subantarctic copepod Neocalanus tonsus. *Limnology and oceanography* **32**, 1317-1330.

[90] DeMartini, E.E., DiNardo, G.T. & Williams, H.A. 2003 Temporal changes in population density, fecundity, and egg size of the Hawaiian spiny lobster (Panulirus marginatus) at Necker Bank, Northwestern Hawaiian Islands. *Fishery Bulletin* **101**, 22-31.

[91] DeMartini, E. & Williams, H. 2001 Fecundity and egg size of Scyllarides squammosus (Decapoda: Scyllaridae) at Maro Reef, Northwestern Hawaiian Islands. *Journal of Crustacean Biology* **21**, 891-896.

[92] McEdward, L.R. & Chia, F.-S. 1991 Size and energy content of eggs from echinoderms with pelagic lecithotrophic development. *Journal of Experimental Marine Biology and Ecology* **147**, 95-102. (doi:https://doi.org/10.1016/0022-0981(91)90039-Y).

[93] McEdward, L.R. & Carson, S.F. 1987 Variation in egg organic content and its relationship with egg size in the starfish *Solaster stimpsoni*. *Marine Ecology Progress Series* **37**, 159-169.

[94] McEdward, L.R. & Coulter, L.K. 1987 Egg volume and energetic content are not correlated among sibling offspring of starfish: Implications for life-history theory. *Evolution* **41**, 914-917. (doi:doi:10.1111/j.1558-5646.1987.tb05865.x).

[95] Thompson, R. 1983 The relationship between food ration and reproductive effort in the green sea urchin, Strongylocentrotus droebachiensis. *Oecologia* **56**, 50-57.
